# Performance of a two-item screening for household food insecurity during WIC services

**DOI:** 10.1186/s12889-025-24477-3

**Published:** 2025-08-27

**Authors:** Christopher E. Anderson, M. Pia Chaparro, Shannon E. Whaley

**Affiliations:** 1 Division of Research and Evaluation, Public Health Foundation Enterprises WIC Program, a Program of Heluna Health, City of Industry, CA 91746 USA; 2 Department of Health Systems and Population Health, University of Washington School of Public Health, Seattle, WA 98195 USA; 3 Division of Research and Evaluation, Public Health Foundation Enterprises WIC Program, 13181 Crossroads Pkwy N #540, City of Industry, CA 91746 USA

**Keywords:** WIC, Food security, Screening, Surveillance, Safety-net systems, Validation

## Abstract

**Background:**

The Special Supplemental Nutrition Program for Women, Infants, and Children (WIC) in California introduced household food insecurity (HFI) screening during eligibility certifications in 2019. This study was conducted to evaluate the screening performance of the 2-item Hunger Vital Sign ™ Screener (HVSS2) for HFI during WIC eligibility certification visits in Los Angeles County (LAC), California.

**Methods:**

Study participants completed a 6-item USDA Household Food Security Survey Module (HFSSM6) assessment in *either* the 2020 LAC WIC Survey (July-December 2021) *or* the longitudinal WIC Cash Value Benefit Study (April 2021-May 2022) *and* had WIC administrative HVSS2 screening data within 30 days of HFSSM6 measurement (*n* = 1,113). HVSS2 performance (sensitivity, specificity, positive predictive value, negative predictive value) for detecting HFI (low or very low food security) was evaluated against the HFSSM6.

**Results:**

Nearly one-third (31.1%) of LAC WIC Survey respondents and one-half (50.5%) of WIC Cash Value Benefit Study respondents had HFI based on the HFSSM6, while 15.7% and 20.0% had HFI based on HVSS2 for the two studies, respectively. The HVSS2 had high specificity (0.90–0.93), moderate-to-high negative predictive value (0.54–0.74), low sensitivity (0.29–0.38), and moderate-to-high positive predictive value (0.57–0.83) for HFI.

**Conclusions:**

HVSS2 screening during WIC services has moderate-to-high negative (i.e., screen negative, are food secure) and positive (i.e., screen positive, are food insecure) predictive values for HFI. Dedicated staff training may be needed to improve the performance of the HVSS2 and to make HVSS2 data collected during WIC certifications more helpful in identifying and referring families experiencing HFI to more services.

**Supplementary Information:**

The online version contains supplementary material available at 10.1186/s12889-025-24477-3.

## Background

Food insecurity is a socioeconomic condition in which an individual or household has limited access to sufficient quantity and adequate quality food, primarily driven by limited financial resources [1, 2]. Household food security ranges from high, to marginal, low, and very low food security, with increasingly severe compromises to the quality and quantity of food available to meet the needs of household members [1]. Household food security is associated with diet quality [3–5], obesity [6, 7], and diet-related chronic health conditions [8, 9]. In 2022, 12.8% of households in the United States (US) were food insecure [10]. The prevalence of household food insecurity varies with economic metrics, including the rate of unemployment [11], and is more common among households that are low-income, headed by a single female, or headed by an individual of a racial/ethnic minority [12].

The Special Supplemental Nutrition Program for Women, Infants, and Children (WIC) is a nutrition assistance program managed by the US Department of Agriculture (USDA) that serves pregnant and postpartum women living in low-income households, in addition to their infants and children under age 5 years, with the aim of supporting healthy diets during the critical periods of pregnancy, the postpartum period, and early childhood [13]. WIC served 6.2 million participants monthly in 2021 [14], providing 4 core services including supplemental food packages redeemable for specific healthy foods at approved vendors, nutrition education, breastfeeding support, and health and social service referrals [15]. Cross-sectional evaluations have found higher prevalence of food insecurity among WIC participating households [12]; however, rigorous evaluations correcting for self-selection of food insecure households into WIC have found WIC participation to be associated with lower food insecurity rates [16], while longitudinal evaluations have reported that more consistent participation is also associated with lower rates of food insecurity [17].

WIC aims to support healthy diets among individuals in low-income households, and household food insecurity is an important indicator of nutritional risk in this population [3]. High-quality information about the food security status of WIC-participating households during service provision could help the program better tailor referrals to other nutrition assistance programs and social services, and ensure that families with greater need of additional support are made aware of those resources. To that end, California WIC agencies began to conduct screenings for household food insecurity in 2019 using the 2-item Hunger Vital Sign ™ Screener (HVSS2) [18]. Given higher rates of food insecurity among WIC-participating households [12] and research that indicates transition between household food insecurity severity categories [19] and persistent household food insecurity are more strongly associated with adverse children’s nutritional and growth outcomes [20], the ability to identify patterns of persistence and changes in household food insecurity may be critical to the WIC program maximizing its positive nutritional impacts [17]. The goal of this study was to evaluate the screening performance of the HVSS2 within the context of WIC service delivery, both cross-sectionally and longitudinally, for identifying household food insecurity among WIC-participating households in Los Angeles County, California in 2020–2022, against the USDA 6-item Household Food Security Survey Module (HFSSM6) [21].

## Methods

### Setting and sample

This study includes 2 samples of WIC-participating households in Los Angeles County, California. The first sample includes respondents to the 2020 Los Angeles County WIC Survey (*n* = 6,753), for which the survey instrument is publicly available [22]. Randomly-selected WIC-participating households in Los Angeles County, California were interviewed and received a 10 US dollar (USD) incentive for participating. The survey response rate was 53%. Given the small number of pregnant respondents to the survey, and in an effort to reduce the potential for bias introduced by change in household composition upon delivery, the sample was restricted to surveys for families with children who also had administrative data on household food security collected within the 30 days before or the 30 days after the date of the survey completion (*n* = 634). The second sample includes participants of the WIC Cash Value Benefit Augment Study, a longitudinal study of the associations between an increase to the value of the WIC cash value benefit for fruits and vegetables that was introduced in June 2021 with household food insecurity and child diet (*n* = 3,000) [23]. This second sample was restricted, at each of the three time points (April/May 2021, August/September 2021, April/May 2022), to households with children who also had administrative data on household food security collected within the 30 days before or the 30 days after the date of the survey completion (*n* = 479). The core survey instrument used for the three time-points can be found in Supplemental File 1. The sampling frames for the two studies differed in that the 2020 Los Angeles County WIC Survey drew a random sample of all WIC-participating households in the county, while the 2021–2022 WIC Cash Value benefit study recruited all WIC-participating families with a child age 1–4 years served at 7 WIC sites in Los Angeles County. Both the 2020 Los Angeles County WIC Survey and the WIC Cash Value Benefit Augment Study received Institutional Review Board approval following a full review from the California Health and Human Services Agency Committee for the Protection of Human Subjects, acting in its capacity as an institutional review board. Oral and written informed consent were obtained for respondents to the Los Angeles County WIC Survey and the WIC Cash Value Benefit Augment Study, respectively.

### Food security measures

The 2020 Los Angeles County WIC Survey and the 2021–2022 WIC Cash Value Benefit Augment Study included the USDA HFSSM6 for the prior 12 months and prior 30 days, respectively [21]. HFSSM6 is a validated subset of the 18-item Household Food Security Survey Module, which is used to estimate the prevalence of household food insecurity in the United States in the Current Population Survey [21]. Household food insecurity was categorized based upon the number of affirmative responses to HFSSM6 as food secure (high or marginal food security: 0–1 affirmative responses) or food insecure (low food security: 2–4 affirmative responses; or very low food security: 5–6 affirmative responses) [21].

WIC administrative data collected from 2019 to the present contain the responses to the two items in the HVSS2 [18], assessed during each annual eligibility certification visit. The adult family representative who completes the eligibility certification, generally the primary caregiver of a WIC-participating child, provided responses (never true, sometimes true, often true) to the two items in the screening, including “Within the past 12 months you worried whether your food would run out before you got the money to buy more,” and “Within the past 12 months the food you bought just didn’t last and you didn’t have money to get more.” An affirmative response to either question (sometimes true, often true) is used to categorize a family as food insecure. Negative responses to both questions (never true) are used to categorize a family as food secure [21].

## Other variables

WIC administrative data variables used to characterize the study population included child age (years, continuous), sex, and race/ethnicity-language preference (Asian, Black, English-speaking Hispanic, Spanish-speaking Hispanic, White); maternal educational attainment (< high school completion, high school completion, >high school completion); and household Supplemental Nutrition Assistance Program (SNAP) participation (yes, no) and income (< 50% FPL (federal poverty level), 50 to < 100% FPL, 100 to < 185% FPL).

### Statistical analysis

The sample was characterized by household food security status determined with the HVSS2, separately for the 2020 Los Angeles County WIC Survey (LACWS) and for the first assessment for each child in the WIC Cash Value Benefit Study (CVB), using frequencies and percentages for categorical covariates or means and standard deviations for continuous covariates. Child, maternal, and household characteristics were compared between food secure and food insecure households, determined with the HVSS2, with chi-square tests (categorical variables) or t-tests (continuous variables). Fisher’s exact test was used for variables with multiple small cell sizes (maternal educational attainment in CVB sample; child race/ethnicity in LACWS and CVB samples).

Responses to each of the HVSS2 questions were tabulated separately for each sample and expressed as the frequency and percentage in each response category. Respondents to the longitudinal WIC Cash Value Benefit Study with appropriately timed (within 30 days of one another) HVSS2 data and HFSSM6 data at two or more study time-points (*n* = 260) were included. Patterns of dichotomized food insecurity were determined separately for HVSS2 and HFSSM6 data. Patterns were then combined into a single dichotomous variable (stable food security/improving food security, stable food insecurity/worsening food security). The cross-sectional performance of the HVSS2 for identifying household food insecurity against HFSSM6 was evaluated with (1) sensitivity (the probability of a family screening positive for food insecurity with the HVSS2 when they have food insecurity using the HFSSM6), (2) specificity (the probability of screening negative (i.e., food secure) with the HVSS2 when they do not have food insecurity using the HFSSM6), (3) positive predictive value (the probability that a family with a positive food insecurity screening using the HVSS2 also has a positive food insecurity result using the HFSSM6), and (4) negative predictive value (the probability that a family with a negative food insecurity screening (i.e., food secure) using the HVSS2 also have a negative food insecurity result using the HFSSM6) [24, 25].

The longitudinal performance of the HVSS2 for identifying stable food insecurity/worsening food security against the HFSSM6 was evaluated with sensitivity, specificity, positive predictive value, and negative predictive value. Sensitivity, specificity, positive predictive value, and negative predictive values were calculated for individuals with administrative and survey household food insecurity assessments within 30 days of each other and for household food insecurity status as determined from the HFSSM6: food insecure (low or very low food security) versus food secure (high or marginal food security). To quantify the magnitude of the association between household food insecurity and a positive HVSS2 screening, odds ratios (OR) and 95% confidence intervals (CI) were calculated with logistic regression analyses for the odds of a positive HVSS2 screening associated with household food insecurity determined by HFSSM6. All analyses were conducted using SAS 9.4 [26], and p-values < 0.05 were considered statistically significant.

## Results

Nearly 16% of children in the Los Angeles County WIC Survey sample (hereafter called “LACWS sample”) and 20.0% of children in the WIC Cash Value Benefit Study sample (hereafter called “CVB sample”) lived in a food insecure household as determined by the HVSS2 (Table 1). Most children in both samples were English-speaking (38.3% and 56.8%) or Spanish-speaking (35.2% and 23.2%) Hispanic. No significant differences between food secure and food insecure households were observed for maternal education and household SNAP participation. In the LACWS sample, English-speaking Hispanic children were a larger proportion of those in food secure households (36.9% vs. 26.0%) and White children were a larger proportion of those in food insecure households (10% vs. 3.4%) (p-value 0.006). Male children were a larger proportion of children in food insecure households (57.0% and 60.4%) compared to food secure households (44.9% and 47.3%) in both LACWS and CVB samples, respectively (p-values 0.03 and 0.02). Households with income < 50% FPL represented a larger proportion of food insecure compared to food secure households (35.4% vs. 22.2%) in the CVB sample (p-value 0.03).

**Table 1:** Description of children included in the 2020 Los Angeles County WIC Survey or the 2021–2022 WIC Cash Value Benefit Study who also had administrative data on food security status collected with the Hunger Vital Sign ™ screener within 30 days of study assessment of household food security (*n* = 1,113)

Characteristic		2020 LA county WIC survey sample			WIC cash value benefit study sample^b^		
		Food secure^a ^*n* = 534	Food insecure^a ^*n* = 100	p-value^c^	Food secure^a ^*n* = 383	Food insecure^a ^*n* = 96	p-value^c^
Male		240 (44.9)	57 (57.0)	0.03	181 (47.3)	58 (60.4)	0.02
Child age, years (Mean ± SD)		2.53 ± 1.17	2.39 ± 1.20	0.28	2.8 ± 1.09	2.95 ± 1.12	0.24
Race/ethnicity				0.006			0.07
Asian		69 (12.9)	12 (12.0)		11 (2.9)	5 (5.2)	
Black		44 (8.2)	15 (15.0)		38 (9.9)	10 (10.4)	
Hispanic, Spanish-speaking		197 (36.9)	26 (26.0)		82 (21.4)	29 (30.2)	
Hispanic, English-speaking		206 (38.6)	37 (37.0)		229 (59.8)	43 (44.8)	
White		18 (3.4)	10 (10.0)		14 (3.7)	5 (5.2)	
Other		0 (0)	0 (0)		9 (2.3)	4 (4.2)	
Maternal education				0.50			0.69
< High school completion		143 (26.8)	28 (28.0)		20 (5.2)	5 (5.2)	
High school completion		151 (28.3)	33 (33.0)		37 (9.7)	11 (11.5)	
>High school completion		240 (44.9)	39 (39.0)		326 (85.1)	80 (83.3)	
Participate in SNAP		252 (47.2)	46 (46.0)	0.83	84 (21.9)	30 (31.3)	0.06
Household income				0.60			0.03
< 50% FPL		114 (21.3)	25 (25.0)		85 (22.2)	34 (35.4)	
50 to < 100% FPL		247 (46.3)	47 (47.0)		142 (37.1)	31 (32.3)	
≥ 100% FPL		173 (32.4)	28 (28.0)		156 (40.7)	31 (32.3)	
USDA 6-item food security				< 0.0001			< 0.0001
High/marginal		394 (73.8)	43 (43.0)		221 (57.7)	16 (16.7)	
Low		119 (22.3)	45 (45.0)		111 (29.0)	35 (36.5)	
Very low		21 (3.9)	12 (12.0)		51 (13.3)	45 (46.9)	

Abbreviations: FPL, federal poverty level; HVSS2, 2-item Hunger Vital Sign ™ Screener; SD, standard deviation; SNAP, Supplemental Nutrition Assistance Program; USDA, United States Department of Agriculture; WIC, the Special Supplemental Nutrition Program for Women, Infants, and Children;

^a^Food insecurity was determined as an affirmative response (“sometimes true” or “often true”) to either of the HVSS2 questions: “Within the past 12 months you worried whether your food would run out before you got the money to buy more,” and “Within the past 12 months the food you bought just didn’t last and you didn’t have money to get more.”

^b^For the sample from the longitudinal WIC Cash Value Benefit Study, all characteristics were determined for the first available time point for each participant

^c^P-values were from chi-square tests of independence for categorical variables, or t-tests for continuous variables. Maternal educational attainment (Sample 2) and child race/ethnicity (Sample 1 and Sample 2) were evaluated with Fisher’s exact test

The HFSSM6 identified nearly one-third of children as living in a food insecure household (25.9% low food security, 5.2% very low food security) in the LACWS sample, and identified half of children as living in a food insecure household (30.5% low food security, 20.0% very low food security) in the CVB sample. There was a strong association between the HVSS2 and HFSSM6 food insecurity status determinations in both samples (both p-values < 0.0001). Categories of food insecurity (low + very low food security) according to the HFSSM6 were more common among households that were food insecure according to the HVSS2 (45.0% and 12.0% for low and very low, respectively, in the LACWS sample; 36.5% and 46.9% for low and very low, respectively, in the CVB sample) than among households that were food secure according to the HVSS2 (22.3% and 3.9% for low and very low, respectively, in the LACWS sample; 29.0% and 13.3% for low and very low, respectively, in the CVB sample). Among LACWS sample respondents, families with low and very low food security determined by HFSSM6 had 3.47 times (OR 3.47, 95% CI 2.18, 5.52) and 5.24 times (OR 5.24, 95% CI 2.41, 11.38) higher odds of a positive food insecurity screening with HVSS2, respectively, than families with high/marginal food security according to HFSSM6 (data not shown). Among CVB sample respondents, families with low and very low food security determined by HFSSM6 had 4.08 times (OR 4.08, 95% CI 2.19, 7.61) and 11.42 times (OR 11.42, 95% CI 6.05, 21.56) higher odds of a positive food insecurity screening with HVSS2, respectively, than families with high/marginal food security according to HFSSM6 (data not shown).

Response distributions for the questions in the HVSS2 are presented in Table 2. A small proportion of households indicated that they often or sometimes worried their food would run out before they got money to buy more for each sample and at all time points (0.7–1.6% often, 12.2–17.0% sometimes), and the majority indicated that this was never the case (81.8–86.3%). Similarly, a small proportion of households expressed that the food they bought just didn’t last and they didn’t have money to buy more often or sometimes (0.7–1.3% often, 12.2–14.6% sometimes), while the majority indicated this was never the case (84.7–86.7%).

**Table 2:** Response frequencies to HVSS2 among WIC-participating households in Los Angeles County, California (*n* = 1,113)

HVSS2 items		Sample^a^	Often true	Sometimes true	Never true
			*N* (%)	*N* (%)	*N* (%)
Within the past 12 months…					
1. …you worried whether your food would run out before you got money to buy more.		LA County WIC Survey sample	10 (1.6)	77 (12.2)	547 (86.3)
		WIC CVB Study sample, time 1	5 (1.3)	66 (17.0)	318 (81.8)
		WIC CVB Study sample, time 2	2 (0.7)	47 (15.6)	253 (83.8)
		WIC CVB Study sample, time 3	2 (1.1)	27 (14.9)	152 (84.0)
2. …the food you bought just didn’t last and you didn’t have money to get more.		LA County WIC Survey sample	8 (1.3)	89 (14.0)	537 (84.7)
		WIC CVB Study sample, time 1	3 (0.8)	53 (13.6)	333 (85.6)
		WIC CVB Study sample, time 2	2 (0.7)	44 (14.6)	256 (84.8)
		WIC CVB Study sample, time 3	2 (1.1)	22 (12.2)	157 (86.7)

Abbreviations: HVSS2, 2-item Hunger Vital Sign ™ Screener; WIC, the Special Supplemental Nutrition Program for Women, Infants, and Children.

^a^The LA County WIC Survey was conducted in July-December 2020, the WIC CVB Study time 1 from April-May 2021, the WIC CVB Study time 2 from August-September 2021, and the WIC CVB Study time 3 from April-May 2022.

Longitudinal patterns of administrative (HVSS2) and CVB sample-based (HFSSM6) household food insecurity were summarized (Table 3). Patterns were dichotomized into two groups indicating stable food security/improving food security (78.8% of HVSS2-determined patterns, 56.9% of HFSSM6-determined patterns) or stable food insecurity/worsening food security (21.2% of HVSS2-determined patterns, 43.1% of HFSSM6-determined patterns).

**Table 3:** Distribution of longitudinal food insecurity patterns among respondents to the WIC Cash Value Benefit Study, conducted in Southern California April 2021-May 2022 (*n* = 260)

Pattern grouping	HVSS2 (*n* = 260)	HFSSM6 (*n* = 260)	Food insecurity at each study visit		
			Time 1	Time 2	Time 3
Stable food security/improving food security	205 (78.8)	148 (56.9)	No	No	No
			Yes	No	No
			Yes	Yes	No
			Yes	No	-
			Yes	-	No
			-	Yes	No
Stable food insecurity/worsening food security	55 (21.2)	112 (43.1)	Yes	No	Yes
			No	Yes	Yes
			No	Yes	-
			No	-	Yes
			-	No	Yes
			Yes	Yes	-
			Yes	Yes	Yes
			Yes	-	Yes

Abbreviations: HFSSM6, the USDA 6-item Household Food Security Survey Module; HVSS2, 2-item Hunger Vital Sign™ Screener; WIC, the Special Supplemental Nutrition Program for Women, Infants, and Children.

The performance of the HVSS2 against the HFSSM6 was evaluated cross-sectionally for every data collection point (Table 4). The HVSS2 used during administrative data collection was found to have low sensitivity and high specificity for food insecurity in all time points (sensitivity: 0.29–0.34; specificity: 0.90–0.93). Predictive values of a negative HVSS2 screening (i.e., negative predictive value) was high for food insecurity among the LACWS sample (0.74), while the predictive value of negative HVSS2 screening was moderate at times 1–3 of the CVB sample (0.54–0.67). The predictive value of a positive screening (i.e., positive predictive value) was moderate for food insecurity among the LACWS sample (0.57), and high at times 1–3 the CVB sample (0.75–0.83). In the longitudinal assessment of the CVB sample, HVSS2-determined patterns of food insecurity from WIC administrative screening had low sensitivity and high specificity (0.38 and 0.91, respectively), moderate negative predictive value (0.66) and high positive predictive value (0.76), for identifying stable food insecurity/worsening food security.

**Table 4:** Sensitivity, specificity, and predictive values of positive and negative screening for household food insecurity ^a^ using the HVSS2 ^b^ among WIC-participating households in Los Angeles County, California (*n* = 1,113)

Measure	LA County WIC Survey sample	WIC Cash Calue Benefit Study sample			
	*n* = 634	Time 1*n* = 389	Time 2*n* = 302	Time 3*n* = 181	Longitudinal patterns^c^*n* = 260
Sensitivity	0.29 (0.23, 0.35)	0.30 (0.24, 0.37)	0.34 (0.26, 0.43)	0.30 (0.20, 0.40)	0.38 (0.29, 0.46)
Specificity	0.90 (0.87, 0.93)	0.93 (0.89, 0.97)	0.92 (0.88, 0.96)	0.92 (0.87, 0.97)	0.91 (0.87, 0.96)
Positive predictive value	0.57 (0.47, 0.67)	0.83 (0.74, 0.91)	0.75 (0.64, 0.86)	0.75 (0.60, 0.90)	0.76 (0.65, 0.88)
Negative predictive value	0.74 (0.70, 0.78)	0.54 (0.48, 0.59)	0.67 (0.62, 0.73)	0.62 (0.55, 0.70)	0.66 (0.59, 0.72)

Abbreviations: HVSS2, 2-item Hunger Vital Sign™ Screener; WIC, the Special Supplemental Nutrition Program for Women, Infants, and Children.

^a^Food insecurity was assessed using the USDA 6-item Household Food Security Survey Module, and categorized as food insecure (low or very low food security) and food secure (high or marginal food security).

^b^Food insecurity screening was performed with the HVSS2. An affirmative response (“sometimes true” or “often true”) to either question was considered a positive screening for household food insecurity: “Within the past 12 months you worried whether your food would run out before you got the money to buy more,” and “Within the past 12 months the food you bought just didn’t last and you didn’t have money to get more.”

^c^Longitudinal food insecurity patterns were determined for WIC Cash Value Benefit Study respondents who had a survey with a complete response to the USDA 6-item Household Food Security Screening Module and administrative Hunger Vital Sign ™ Screener within 30-days of one another at a minimum of 2 study points. Administrative and screening patterns were dichotomized (stable food security/improving food security, stable food insecurity/worsening food security) to evaluate the performance of screening for detecting longitudinal patterns.

## Discussion

Food insecurity measured with the HFSSM6 is strongly associated with a positive food insecurity screening using the HVSS2, introduced for household food insecurity screening during WIC certification visits in California in 2019. The specificity (probability of screening negative [i.e., food secure] given that one is food secure on the HFSSM6) of the HVSS2 as administered in WIC services was high. The negative predictive value (the proportion of negative screening results [i.e., food secure] that are also negative on the HFSSM6) was moderate-to-high at the different study time points. Sensitivity (probability of screening positive [i.e., food insecure] given that one is food insecure on the HFSSM6) of the HVSS2 as administered in WIC services was low, and positive predictive value (the proportion of positive screening results [i.e., food insecure] that are food insecure on the HFSSM6) was moderate-to-high at the different study time points. The present study evaluated the performance of the HVSS2 against both the 30-day and 12-month time frame versions of the HFSSM6, and found similar screening performance, suggesting that the instrument time frame does not have a substantial impact on the screening performance of the HVSS2. While the high specificity of the HVSS2 may help WIC to avoid directing referrals and other additional supports to households who are not food insecure, higher sensitivity of food security screening during the administration of WIC services is critical to providing appropriate referrals to other social services and to ensure those in greatest need of additional support are aware of those resources.

The concordance between the results of the HVSS2 and the HFSSM6, except for lower sensitivity, identified in this study extends prior research showing adequate performance of HVSS2 as a food insecurity screening tool in health care contexts, particularly pediatric care [18, 27], to WIC services. A prior study conducted in a pediatric dental setting identified high sensitivity (0.95) and specificity (0.84) of the HVSS2 against the HFSSM6 [28]. The reported sensitivity is much higher than in the present study (0.29–0.34 for food insecurity), and the specificity similar to the present study (0.90–0.93 for food insecurity). Differences in sensitivity may be due to differences in how questions were administered (in person during a dental visit compared to over the phone with WIC staff), or differences in the time between the two assessments (during the same dental visit compared to between 0 and 30 days between assessments). These differences in the timing and method of data collection for the two studies may differentially prime study participants to respond to the two instruments more or less similarly, and could explain the lower HVSS2 sensitivity found in the present study. Another study reported that a single item screener for food insecurity used in a pediatrics practice was found to have moderate sensitivity (0.59) and high specificity (0.87) [29], suggesting that short screening instruments for food insecurity may have consistently high specificity among families with young children.

The positive predictive value of the HVSS2 was moderate-to-high for food insecurity (0.57–0.83). Negative predictive value of the HVSS2 was moderate-to-high for food insecurity (0.54–0.74). Because predictive values are influenced by the prevalence of the condition being screened for [24], these results are expected. The high specificity of the HVSS2 identified in this study, and prior studies [28, 29], contributes to a high negative predictive value of the screening (i.e. the proportion of negative screening tests that do not have food insecurity according the HFSSM6) when performed in a population with a low prevalence of food insecurity (31.1% among the LACWS sample) and a lower negative predictive value when performed in a population with a higher prevalence of food insecurity (50.5% among the CVB sample) [24]. Similarly, the low sensitivity of the HVSS2 identified in this study, in contrast to the high sensitivity reported in a prior study [28], leads to a moderate positive predictive value (i.e. the proportion of positive screening tests that have food insecurity according to the HFSSM6) when performed in a population with a moderate prevalence of food insecurity (LACWS sample) and higher positive predictive value when performed in a population with a higher prevalence of food insecurity (CVB sample) [24]. In the LACWS sample, a small proportion of families who screened as food secure with the HVSS2 were identified as having very low food security with the HFSSM6 (3.9%), while in the CVB sample a higher proportion of those who screened as food secure with the HVSS2 were identified as having very low food security with the HFSSM6 (13.3%). This could be attributable to participants being more hesitant to report food insecurity to WIC staff during WIC services than to research staff over the phone (LACWS sample) or via an online data collection instrument (CVB sample).

Patterns of household food insecurity identified across the three time-points of the WIC Cash Value Benefit Study (between April 2021 and May 2022) found that over half of households had stable or improving food security (78.8% of households using HVSS2-derived patterns, 56.9% of households using HFSSM6-derived patterns). The screening performance of the HVSS2 for identifying longitudinal patterns of food insecurity was comparable to the cross-sectional assessments at all study time points (low sensitivity, high specificity, high positive predictive value, moderate negative predictive value). This finding is important, because household food insecurity varies in both severity and persistence, with relatively few households in the general US population experiencing persistent food insecurity [30, 31]. Given the much higher prevalence of food insecurity among WIC-participating households than non-participating households [12] and prior research that has reported that periods of transition between household food insecurity severity categories [19] and persistent household food insecurity may be acutely detrimental to children’s nutritional and growth outcomes [20], the ability to identify patterns of persistence and changes in household food insecurity may be critical to the WIC program maximizing its positive nutritional impacts [17].

This study has numerous strengths, including data from a randomly sampled survey and a longitudinal study that recruited all study-eligible individuals. The LA County WIC Survey is a large, recurrent study using a randomly selected sampling frame of WIC-participating households in LA County. Both the cross-sectional and the longitudinal studies included high quality food insecurity assessments with the HFSSM6. Survey and WIC administrative data allowed detailed characterization of the included households. The study also had limitations. Both measures of food insecurity used in this study are at the household level and therefore cannot be used to definitively identify food insecurity for WIC-participating women or children, only the households of WIC-participating women or children. The 6-item USDA HFSSM, a validated shortened version of the full 18-item HFSSM, was used to reduce survey respondent burden; however, the full 18-item HFSSM would be needed to assess whether the HVSS2 can identify food insecurity for specific individuals within WIC-participating households. Missing data for time 2 and time 3 in longitudinal assessment of food insecurity patterns could have altered how household trajectories were classified, and this assessment would benefit from replication in a population in which both food insecurity instruments are used in tandem at multiple time points. The population served by WIC in Los Angeles County is majority Hispanic, and all are low-income, and therefore generalization of the performance of the HVSS2 identified in this analysis should be done cautiously for demographically distinct populations. All WIC services during the study period were administered remotely due to the COVID-19 pandemic, which could impact the types of households participating in WIC and therefore the generalizability of the results, and it is possible that the performance of household food insecurity screening may differ between in-person and telephone-based assessments.

A qualitative study conducted prior to the COVID-19 pandemic reported that food insecurity screening was generally acceptable to caregivers of young children, and that WIC was the most commonly used resource to address household food insecurity among this population [32]. Given the broad reach of the WIC program among children under age 5 in the US [33, 34], embedding food insecurity screening in WIC services may represent a critical means of increasing the proportion of households with young children receiving food insecurity screenings who can then be appropriately referred to further food and social assistance [35]. Further, given that WIC programs systematically document data at the state-level, which is of great public health utility [36], food insecurity screening as part of WIC services may represent both a way of strengthening referrals to other services to improve household food security and of tracking the prevalence of household food security over time.

## Conclusions

In conclusion, household food insecurity screening during the administration of WIC services could provide the WIC program with necessary information to identify families struggling with food insecurity and refer them to other programs and services for additional assistance. The performance of the HVSS2 for food insecurity screening in WIC, particularly its sensitivity and positive predictive value, might be improved by more rigorous training of staff in how to administer these specific questions. Future evaluations of the screening performance of the HVSS2 during in-person WIC service delivery is needed to understand whether the method of screening during WIC service-delivery (in-person, on telephone) contributes to the performance of the instrument. High quality data will be critical to maximizing the value of the screenings for identifying and referring food insecure households to other nutrition assistance programs and social services. The performance of the HVSS2 in longitudinal and cross-sectional evaluations were similar, and may help the WIC program to identify and refer families with incident or persistent food insecurity to additional sources of support. The strong association between the HVSS2 and HFSSM6 suggest that the HVSS2, as currently implemented in routine WIC services, may be useful for research purposes. However, future research is needed to determine whether the screening performance of the HVSS2 varies by household sociodemographic characteristics. This is needed to ensure that screening and additional support are not missing households with specific characteristics (e.g. non-English language preference), and that using the HVSS2 for research does not generate biased results. Food insecurity screening data from WIC participants may also be useful for public health efforts around food insecurity surveillance given the broad reach of the WIC program and the nearly continuous administration of screenings.

## Supplementary Information

Below is the link to the electronic supplementary material.

Supplementary Material 1

## Data Availability

The data described in the manuscript will not be made available because they include confidential administrative data of the WIC program. The code book and analytic code will be made available upon request.

## Abbreviations

CI: Confidence interval
COVID-19: Coronavirus disease-2019
CVB: Cash Value Benefit
FPL: Federal poverty level
HFI: Household food insecurity
HFSSM6: Household Food Security Survey Module
HVSS2: Hunger Vital Sign™ Screener
LACWS: Los Angeles County WIC Survey
OR: Odds ratio
US: United States of America
USD: United States Dollar
USDA: United States Department of Agriculture
SNAP: The Supplemental Nutrition Assistance Program
WIC: The Special Supplemental Nutrition Program for Women, Infants, and Children

## Declarations

### Data, materials, and code availability

The data described in the manuscript will not be made available because they include confidential administrative data of the WIC program. The code book and analytic code will be made available upon request.
