# Supplementary material for: Performance of a two-item screening for household food insecurity during WIC services
Source: BMC Public Health. 2025 Aug 27;25:2959. doi: 10.1186/s12889-025-24477-3 (PMC12382218; doi:10.1186/s12889-025-24477-3)
Supplement: Supplementary file 1 — Supplementary Material 1 [file 12889_2025_24477_MOESM1_ESM.docx]

PHFE WIC is inviting you to complete a survey about your child’s food consumption and food access. Your responses will help WIC provide the best services possible. Completing this survey is voluntary and your responses will not impact your WIC benefits, but your responses are very important to us. The survey will take less than 10 minutes and you can skip any questions that you do not want to answer. We will not be asking for your name or contact information and will not be sharing any private information about you with others.

If you have any questions you can contact [RESEARCH STAFF NAME] at [PHONE NUMBER].

The first two questions are about the **fresh fruits and vegetables in your community**. Community is the neighborhood where you live, and other neighborhoods that you are easily able to get to. **Please select how much you agree or disagree with each statement.**

|  | **Strongly agree** | **Agree** | **Neither agree nor disagree** | **Disagree** | **Strongly disagree** |
| --- | --- | --- | --- | --- | --- |
| 1. It is easy to buy fresh fruits and vegetables in my community | 󠅂 | 󠅂 | 󠅂 | 󠅂 | 󠅂 |
| 1. The fresh fruits and vegetables in my community are of high quality | 󠅂 | 󠅂 | 󠅂 | 󠅂 | 󠅂 |

The next questions are about the food eaten in your household in the past month, that is, the **past 30 days**. **During the past month, select how often each of the following statement was true for YOUR HOUSEHOLD.**

| 1. The food that we bought just didn’t last, and we didn’t have money to get more. | **Often true 󠅂**  **󠅂** | **Sometimes true**  **󠅂** | **Never true**  **󠅂** |
| --- | --- | --- | --- |
| 1. We couldn’t afford to eat balanced meals. | **Often true 󠅂**  **󠅂** | **Sometimes true**  **󠅂** | **Never true**  **󠅂** |
| Did you or other adults in your household ever cut the size of your meals or skip meals because there was wasn’t enough money for food? | **Yes**  **󠅂** | **No**  **󠅂** | **Don’t know**  **󠅂** |
| 5a. [SKIP if 5=no, don’t know] In the last 30 days, how many days did this happen? | **______ days** |  |  |
| 1. Did you ever eat less than you felt you should because there wasn't enough money for food? | **Yes**  **󠅂** | **No**  **󠅂** |  |
| 1. Were you ever hungry but didn't eat because there wasn't enough money for food? | **Yes**  **󠅂** | **No**  **󠅂** |  |

| 1. How many of the people in your household are under 18 years old? | **1**  **󠅂** | **2**  **󠅂** | **3+**  **󠅂** |
| --- | --- | --- | --- |

Please select a child between the ages of 1 to 4 years old who is on WIC. If you have more than one child between the ages of 1 and 4 on WIC, please select **your youngest child on WIC**. **Do not include an infant less than 12 months**.

| 1. What is the birthdate of your **youngest child on WIC (between the ages of 1 and 4 years)**? | **Month**  **(drop down Jan-Dec)** | **Day**  **(drop down 1-31)** | **Year**  **󠅂2017 󠅂2018**  **󠅂2019**  **󠅂2020** |
| --- | --- | --- | --- |
| 1. Is your **youngest child on WIC (between the ages of 1 and 4 years)** a girl or boy? | Girl  󠅂 | Boy  󠅂 |  |

We would like to know about the foods your **youngest child on WIC between the ages 1 and 4 years old** ate or drank the **past month**, that is, the past **30 days**. When answering, please include meals and snacks at home, at childcare, in restaurants or take out, and any other place. You can answer never or the number of times per month, per week or per day. **During the past month, select how often YOUR youngest CHILD on WIC between the ages of 1 and 4 years ate each of the following. Please select only one response for each question.**

| **During the past month (30 days):** | **Never** | **1**  time last **month** | **2-3** times last **month** | **1**  time per **week** | **2** times per **week** | **3-4** times per **week** | **5-6** times per **week** | **1**  time per **day** | **2-3** times per **day** | **4 or more** times per **day** |
| --- | --- | --- | --- | --- | --- | --- | --- | --- | --- | --- |
| 1. **100% pure fruit juices** such as orange, mango, apple, grape and pineapple juices [Do **not** include fruit-flavored drinks with added sugar or fruit juice you made and added sugar to] | 󠅂 | 󠅂 | 󠅂 | 󠅂 | 󠅂 | 󠅂 | 󠅂 | 󠅂 | 󠅂 | 󠅂 |
| 1. **Fruit**. Include fresh, frozen or canned fruit [Do **not** include juices] | 󠅂 | 󠅂 | 󠅂 | 󠅂 | 󠅂 | 󠅂 | 󠅂 | 󠅂 | 󠅂 | 󠅂 |
| 1. Green leafy or lettuce **salad**, with or without other vegetables | 󠅂 | 󠅂 | 󠅂 | 󠅂 | 󠅂 | 󠅂 | 󠅂 | 󠅂 | 󠅂 | 󠅂 |
| 1. Any kind of **fried potatoes**, including french fries, home fries, or hash brown potatoes | 󠅂 | 󠅂 | 󠅂 | 󠅂 | 󠅂 | 󠅂 | 󠅂 | 󠅂 | 󠅂 | 󠅂 |
| 1. Any **other kind of potatoes**, such as baked, boiled, mashed potatoes, sweet potatoes, or potato salad | 󠅂 | 󠅂 | 󠅂 | 󠅂 | 󠅂 | 󠅂 | 󠅂 | 󠅂 | 󠅂 | 󠅂 |
| 1. Refried beans, baked beans, beans in soup, pork and beans or any other type of cooked dried beans [Do **not** include green beans] | 󠅂 | 󠅂 | 󠅂 | 󠅂 | 󠅂 | 󠅂 | 󠅂 | 󠅂 | 󠅂 | 󠅂 |
| 1. **Other vegetables** [Do **not** include green salads, potatoes, cooked dried beans] | 󠅂 | 󠅂 | 󠅂 | 󠅂 | 󠅂 | 󠅂 | 󠅂 | 󠅂 | 󠅂 | 󠅂 |
| 1. Mexican-type **salsa** made with tomato | 󠅂 | 󠅂 | 󠅂 | 󠅂 | 󠅂 | 󠅂 | 󠅂 | 󠅂 | 󠅂 | 󠅂 |
| 1. **Pizza**. Include frozen pizza, fast food pizza, and homemade pizza | 󠅂 | 󠅂 | 󠅂 | 󠅂 | 󠅂 | 󠅂 | 󠅂 | 󠅂 | 󠅂 | 󠅂 |
| 1. **Tomato sauces** such as with spaghetti or noodles or mixed into foods such as lasagna [Do **not** include tomato sauce on pizza] | 󠅂 | 󠅂 | 󠅂 | 󠅂 | 󠅂 | 󠅂 | 󠅂 | 󠅂 | 󠅂 | 󠅂 |
| 1. **Regular soda or pop** that contains sugar? [Do **not** include diet soda] | 󠅂 | 󠅂 | 󠅂 | 󠅂 | 󠅂 | 󠅂 | 󠅂 | 󠅂 | 󠅂 | 󠅂 |
| 1. **Sweetened** fruit drinks, sports or energy drinks, such as KoolAid, lemonade, HiC, cranberry drink, Gatorade, Red Bull or Vitamin Water? Include fruit juices you made at home and added sugar to. [Do **not** include diet drinks or artificially sweetened drinks] | 󠅂 | 󠅂 | 󠅂 | 󠅂 | 󠅂 | 󠅂 | 󠅂 | 󠅂 | 󠅂 | 󠅂 |
| 1. **Chocolate** or any other types of candy? [Do **not** include sugarfree candy] | 󠅂 | 󠅂 | 󠅂 | 󠅂 | 󠅂 | 󠅂 | 󠅂 | 󠅂 | 󠅂 | 󠅂 |
| 1. **Doughnuts**, sweet rolls, Danish, muffins, pan dulce, or poptarts? [Do **not** include sugarfree items] | 󠅂 | 󠅂 | 󠅂 | 󠅂 | 󠅂 | 󠅂 | 󠅂 | 󠅂 | 󠅂 | 󠅂 |
| 1. **Cookies, cake, pie or brownies**? [Do **not** include sugarfree kinds] | 󠅂 | 󠅂 | 󠅂 | 󠅂 | 󠅂 | 󠅂 | 󠅂 | 󠅂 | 󠅂 | 󠅂 |
| 1. **Ice cream or other frozen desserts**? [Do **not** include sugarfree kinds] | 󠅂 | 󠅂 | 󠅂 | 󠅂 | 󠅂 | 󠅂 | 󠅂 | 󠅂 | 󠅂 | 󠅂 |
| 1. **Food from a fast food restaurant** such as McDonald's, Taco Bell, or KFC | 󠅂 | 󠅂 | 󠅂 | 󠅂 | 󠅂 | 󠅂 | 󠅂 | 󠅂 | 󠅂 | 󠅂 |

| 1. Families with a child ages 1 through 4 years old receive $9 per month from WIC to purchase fruits and vegetables for that child. What do you think about the $9 amount given for fruits and vegetables? Would you say it is: | Too much  󠅂 | Not enough  󠅂 | | Just right  󠅂 | | Don’t know 󠅂 󠅂 | |
| --- | --- | --- | --- | --- | --- | --- | --- |
| 29. Do you consent for your survey answers to be used for research?  (Note: your answer will not impact your WIC benefits). | | | **Yes**  **󠅂** | | **No**  **󠅂** | |  |
| 30. Please enter the last 10 digits of your California WIC Card number: XXXX XX_ _ _ _ _ _ _ _ _ _ | | | | | | |  |

**Thank you for completing this survey!**
